# Prevalence and exploratory factor analysis of long COVID-19 symptoms among experienced infected population in Bangkok, Thailand

**DOI:** 10.1186/s12889-024-20275-5

**Published:** 2024-10-17

**Authors:** Suphanna Krongthaeo, Suphamas Partiprajak, Noppawan Piaseu, Sineenuch Ckumdee, Chonthicha Taaon, Anon Kongsuwan

**Affiliations:** 1 Ramathibodi School of Nursing, Faculty of Medicine Ramathibodi Hospital, Mahidol University, Rama VI Road, Phaya Thai, Ratchathewi, Bangkok, 10400 Thailand; 2 Nursing Department, Faculty of Medicine Ramathibodi Hospital, Ramathibodi Hospital, Mahidol University, Bangkok, Thailand; 3 Center for Health Promotion and Well-Being, Faculty of Medicine Ramathibodi Hospital, Mahidol University, Bangkok, Thailand

**Keywords:** Long COVID, Prevalence, Symptom cluster, Functional disability, Thailand

## Abstract

**Background:**

Patients with long COVID may experience various concomitant symptoms caused by inflammation, which affect their lives and well-being. In this study, we aimed to (1) investigate the prevalence of long COVID; (2) explore the levels of symptom severity and functional disability owing to long COVID, overall health, and their relationship; and (3) conduct exploratory factor analysis of long COVID-19 symptoms among experienced infected population in the capital of Thailand.

**Methods:**

A cross-sectional research design was used and a sample of 337 community members with previously COVID-19 infection in Bangkok, Thailand was recruited for this study. Purposive sampling was used. Data collection was performed using an online and a paper-based questionnaire. Descriptive statistics (number, percentage), odds ratio, exploratory factor analysis, and Spearman’s rank correlation coefficient were used for the data analysis.

**Results:**

The prevalence of long COVID was 32.9%. The main reported symptoms included anxiety (28.5%), fatigue (26.1%), and dyspnea (13.4%). There was a significant relationship between symptom severity and functional disability (r_s_=0.385, p value < 0.01). Overall health was negatively correlated with symptom severity (r_s_ = − 0.291, *p* < .01) and functional disability (r_s_ = − 0.108, *p* < .05). Using principal component analysis with Promax rotation, three clusters were identified, explaining 71.44% of the total variance. The Clusters comprised (1) common symptoms of long COVID and communication, (2) fatigue, functioning, and nutritional concerns, and (3) psychosocial impacts.

**Conclusions:**

The present results might help multidisciplinary care teams understand the concurrent symptoms of patients with long COVID and develop rehabilitation care programs to ease all symptoms simultaneously and improve patients’ quality of life.

**Supplementary Information:**

The online version contains supplementary material available at 10.1186/s12889-024-20275-5.

## Introduction

COVID-19 remains a public health crisis and has placed an extensive burden on health care systems worldwide. After more than 40 months of the COVID-19 pandemic, SARS-CoV-2 has not yet been eliminated owing to its genomic variation and transmission dynamics. According to the latest COVID-19 statistics from the World Health Organization, as of 11 June 2023, there were 767,750,853 confirmed COVID-19 cases and 6,941,095 deaths [1]. In Thailand, on 11 June 2023, the total number of confirmed cases is 4,745,043 and 34,163 of deaths, respectively [2]. In Bangkok, on 18–24 June 2023, the new cases of COVID-19 infection were 299 and the deaths were 6 [3]. Symptoms of acute illness usually occur from 2 to 14 days after exposure to the virus [4]. The recovery period ranges from 7 to 10 days from the initial onset of symptoms [5]. The prognosis of COVID-19 infection varies from mild or moderate to severe disease depending on age, sex, obesity, and comorbidities [5, 6]. Nevertheless, many COVID-19 survivors retain some symptoms or develop new symptoms after 4 weeks of infection.

The term “long COVID” is widely used to describe signs and symptoms that persist or develop after the acute phase of infection. Long COVID is defined as the continuation or development of new symptoms after 4 weeks of the initial SARS-CoV-2 infection [7]. Long COVID symptoms include both ongoing symptomatic COVID-19 infection (4 to 12 weeks) and post COVID-19 syndrome (> 12 weeks) [7–9]. The symptoms of long COVID encompass a cluster including damage to various tissues, organs, and body systems such as the respiratory, cardiovascular, central nervous, endocrine, urinary, and immune systems. The most common symptoms of long COVID are dyspnea, fatigue, palpitations, dizziness, pain, cognitive and attention impairment, anxiety, depression, post-traumatic stress disorder (PTSD), and allergic reactions. Patients with any severity of acute COVID infection symptoms, even mild cases, can develop long COVID. The risk factors of long COVID are older age, female sex, and underlying health problems [10]. The presence of more than five symptoms during acute infection is also reported as a risk factor [5].

Long COVID symptoms are triggered by the excessive production of pro-inflammatory cytokines, which are potentiated by the production of reactive oxygen species [11, 12] and a dysregulated immune–inflammatory response resulting in central nervous system (CNS)-mediated long COVID symptoms including fatigue, malaise, fever, dyspnea, and cognitive and attention impairment [12]. This could be related to the concept of symptom management [13, 14], which has been extensively researched in patients with cancer. A symptom cluster is defined as three or more concurrent symptoms that are related to each other [13]. Furthermore, symptoms within the same cluster may or may not share a common mechanism or a common etiology. Miaskowski et al. posited that symptoms can be related via a common mechanism, by sharing variance, or by producing different outcomes than individual symptoms [14]. For this reason, investigation of symptom clusters can assist in developing a comprehensive care management program for patients with long COVID, simultaneously encompassing all related symptoms within management. In Thailand, 75.6% of long COVID prevalence has been found. The most frequently reported symptoms were difficulties performing daily activities, breathlessness, and fatigue [15].

Patients with long COVID experience a variety of symptoms that affect their daily activities and quality of life [16]. To date, there is a paucity of evidence related to symptom clusters of long COVID across countries. In Thailand, there are no studies of long COVID in terms of clusters of symptoms and functional ability. We conducted this study in Bangkok because it is the most overcrowded city in Thailand. The incidence of COVID-19 was higher than in other districts. In the Ratchathewi subdistrict, there are more than twenty overcrowded communities with people of low socioeconomic status, limited space, and poor conditions, contributing to a higher impact from long COVID conditions and a lower quality of life.

Therefore, in this study, we aimed to (1) explore the prevalence of long COVID; (2) investigate patients’ severity of symptoms, functional disability, and overall health as well as their relationship; and (3) conduct the exploratory factor analysis of long COVID-19 symptoms among experienced infected population in the capital city of Thailand. This study is crucial for identifying long COVID symptoms so that healthcare providers can establish effective rehabilitation care programs to improve patient’s quality of life.

## Methods

### Design and sample

We used a cross-sectional research design, as indicated. The population was the community members that had experienced COVID-19 infection in Bangkok, Thailand. The sample included 337 community members in Bangkok. The ratio of items to participants was greater than 1:20. Therefore, the sample size was acceptable for exploratory factor analysis [17]. Purposive sampling was used following these inclusion criteria: (1) age greater than 20 years, (2) recovering from COVID-19, and (3) living in Ratchathewi subdistrict, Bangkok. The exclusion criteria were the community members that did not completely respond to all of the items of the questionnaires. The research setting was the communities of Ratchathewi, Bangkok, Thailand.

### Measures

We applied two instruments for collecting participants’ health information and severity of long COVID.

1. A 14-item personal health questionnaire was developed to obtain participants’ demographic data and information on personal health status, initial symptoms, treatment during the acute phase of COVID-19, and ongoing or new symptoms during the post-acute phase. This questionnaire included age, sex, education, occupation, family income, weight, height, past medical history, smoking, symptom categories during acute COVID-19 infection, developed pneumonia during acute COVID, acute care setting, post COVID-19 duration, and symptoms of long COVID.
2. The COVID-19 Yorkshire Rehabilitation Scale (C19-YRS), Thai version, a standardized self-report measure of symptom severity owing to long COVID, was translated into Thai language using forward and back translation, including cross-cultural aspects by Partiprajak et al. The original English version (https://licensing.leeds.ac.uk/product/c19-yrs-covid-19-yorkshire-rehabilitation-scale) was developed by Sivan and a multidisciplinary team of rehabilitation professionals in 2021 [18, 19] and it was translated into Thai by two bilingual translators. Then, the Thai version was blind back-translated into English. After that, the experts reviewed both the original and back-translated versions and then sent them back to the original developer for approval of its appropriateness. Five experts evaluated the content validity (CVI). The CVI was 0.95. The test-retest reliability was tested with 30 participants that had recovered from COVID-19 in Bangkok after 2 weeks. The correlation coefficient was 0.78 [20].

However, the original instrument has been modified based on Rasch analysis, recommendations from patients and health care professionals, and evidence-based practice [21]. In this study, some items regarding major symptoms and functional disability in the original version were eliminated during the validation process for the instrument [20, 21]. The Thai version comprises three subscales, including major symptoms (9 items), functional disability (3 items), and overall health (1 item). Major symptoms include:

- breathlessness at rest,
- cough or throat discomfort,
- voice changes,
- nutritional concerns,
- fatigue,
- pain or discomfort,
- difficulty with concentration,
- difficulty with short-term memory,

i) depression.

The three items of functional disability included (1) communication, (2) mobility, and (3) activities of daily living. A self-rated scale on perceived overall health was also included. For each item, participants answered “Yes” or “No” questions. If the response was “Yes,” participants were asked to rate the severity of symptoms and functional disability, ranging from 0 to 10 and compare the severity between the present time and prior to developing COVID-19 symptoms. The scores for overall health also ranged from 0 to 10, with a higher score indicating greater severity. For each symptom, the score was categorized from mild (0 to 2), moderate (3 to 5), or severe (6 or higher). The total score of major symptoms ranged from 0 to 90, from 0 to 30 for functional disability and from 0 to 10 for overall health. The reliability of the final C19-YRS Thai version was tested on 337 participants in Ratchathewi, Bangkok, Thailand [20]. The Cronbach’s alpha coefficient was 0.723, indicating acceptable reliability.

### Data collection

Data collection was conducted between November 2021 and May 2022, after receiving study approval from the Institutional Review Board Committee on Human Research at Faculty of Medicine Ramathibodi Hospital, Mahidol University. Community leaders in Bangkok were asked to contact their community members who had experienced COVID-19 infection. We used two methods for data collection. All of the participants met directly with the principal investigator (PI). We collected data via online questionnaires by scanning a QR code from the PI so that the participants would be comfortable with Internet access. Data were also collected using paper-based questionnaires at the community meeting room for the participants that could not access the Internet. The time required to complete the questionnaire was approximately 15 min.

### Data analysis

Data analysis was conducted using IBM SPSS for Windows version 21 (IBM Corp., Armonk, NY, USA). Descriptive statistics (number, percentage) were used to analyze participants’ characteristics. Symptom prevalence was analyzed using the odds ratio. Construct validity was investigated using exploratory factor analysis (EFA) with principal component analysis. Oblique rotation with the Promax method was used because it is appropriate for interrelated factors. We hypothesized that the severity of symptoms and functional disability are correlated to a certain extent. Components were extracted based on: (1) a Kaiser–Meyer–Olkin (KMO) value above 0.5, demonstrating sampling adequacy; (2) the significance of Bartlett’s test of sphericity to verify that the correlation matrix is an identity matrix; (3) measure of sampling adequacy (MSA) greater than 0.5 as relevant for factor analysis; (4) eigenvalue more than 1.0 representing the total variance in all items that can be explained by a certain factor; and (5) a cut-point of factor loading above 0.35 for the remaining items. Finally, Spearman rank correlation was used to identify the relationships among factors and the association between the severity of long COVID and overall health.

## Results

### Participants’ characteristics

More than half of participants were women (53.7%). Most (82.5%) participants were aged 21 to 60 years (mean = 45.94, SD = 15.61). Most (79.8%) participants had a primary or secondary education. Nearly one-fourth (23.1%) of participants were unemployed, and 76.3% had family incomes less than 20,000 THB per month. Nearly half of the participants (44.8%) were obese with a BMI > 25 kg/m^2^; 43% had a medical history of underlying diseases. The proportions of patients with hypertension and diabetes were 24.0%, and 11.9%, respectively, and approximately one-third (30.86%) had cardiovascular diseases including cardiac ischemia, myocardial infarction, and stroke. Most of the participants (88.1%) were non-smokers.

Among the total, 88.1% of participants met the criteria for the category of mild symptoms of acute COVID-19 at registration. A total of 17.8% of participants were diagnosed with pneumonia during the acute period of infection. The care settings during acute infection were the field hospital in the community (58.2%) and home isolation (32.9%). The participants’ characteristics are shown in Supplementary Table 1.

**Fig. 1:**
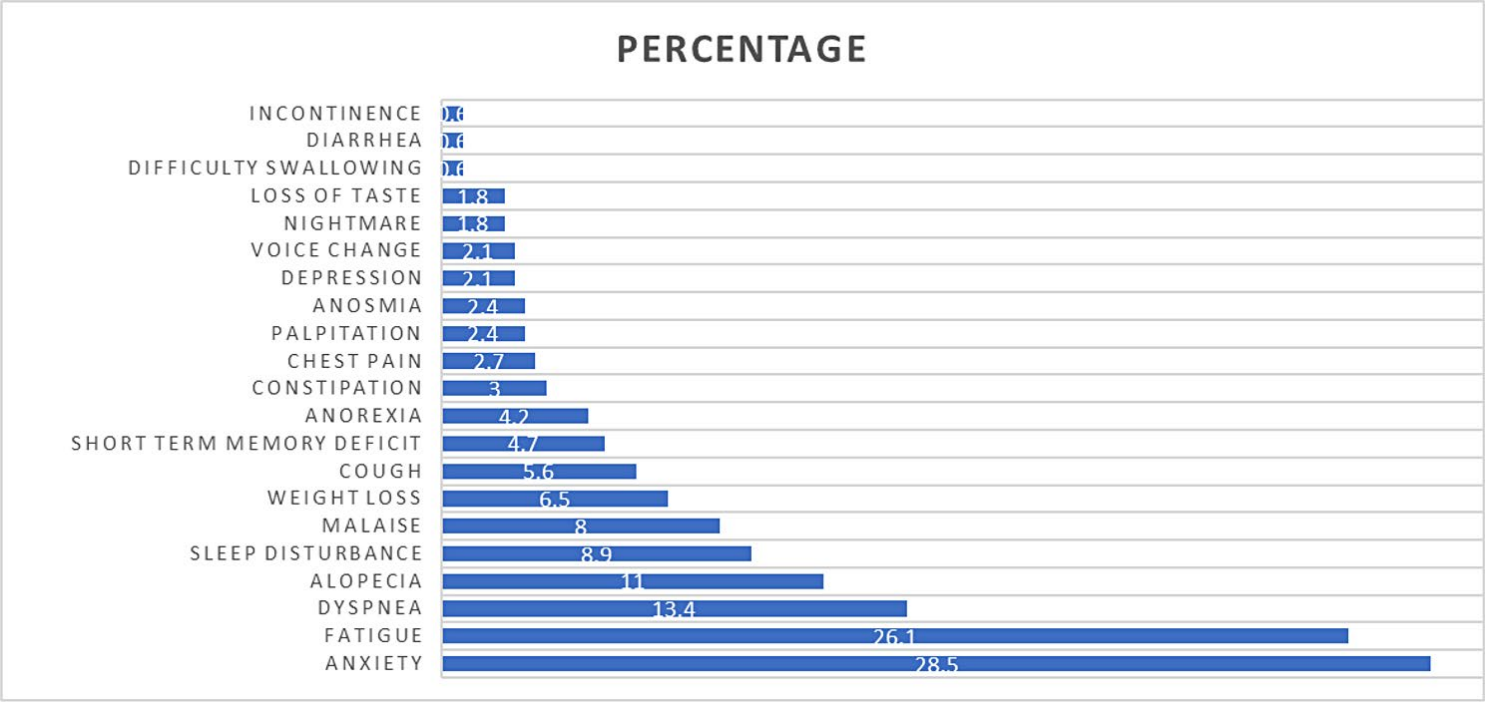
Symptoms of long COVID

### Prevalence of long COVID

The prevalence of long COVID was 32.9% (111 of 337 cases). Long COVID symptoms, illustrated as percentages, are shown in Fig. 1. Anxiety was the most commonly reported symptom, with a prevalence of 28.5%, followed by fatigue (26.1%) and dyspnea (13.4%).

Factors that were significantly associated with the prevalence of long COVID were female sex (odds ratio [OR] = 1.98; 95% confidence interval [CI] = 1.24–3.17), underlying cardiovascular disease (OR = 1.93; 95% CI = 1.19–3.12), obesity (OR = 1.95; 95% CI = 1.23–3.09), and smoking (OR = 0.59; 95% CI = 0.34–0.98). No significant difference was found between the prevalence of long COVID in the older adult and adult groups (Table 1).

**Table 1:** Prevalence of long COVID among Thai community members according to selected participant characteristics (*n* = 337)

Characteristics	*n* (%)	Having long COVID		OR (95% CI)
		No	Yes	
Age				1.51 (0.85–2.68) 1 [reference]
Older adult	59	35	24	
Adult	278	191	87	
Sex				1.98 (1.24–3.17) * 1 [reference]
Female	181	109	72	
Male	156	117	39	
Underlying cardiovascular disease				
Yes	104	59	45	1.93 (1.19–3.12) * 1 [reference]
No	233	167	66	
Obesity				
Yes	151	89	62	1.95 (1.23–3.09) * 1 [reference]
No	186	137	49	
Smoking				0.59 (0.34–0.98) * 1 [reference]
Yes	100	75	25	
No	237	151	86	
Severity of acute COVID				
Severe	325	221	104	2.98 (0.92–9.60) 1 [reference]
Mild/moderate	12	5	7	

* *p* < .05

OR, odds ratio; CI, confidence interval

### Severity of symptoms, functional disability, and overall health

The total scores of the Thai C19-YRS for symptom severity and functional disability ranged from 1 to 55 and 0 to 12, respectively. The range of scores for overall health were between 0 and 10. Among participants, 67.1% reported a symptom severity of 0. Likewise, nearly all participants (94.1%) reported no functional disability; these data had a non-normal distribution. Levels of severity for the main persistent symptoms, functional disability, and overall health are presented in Table 2.

**Table 2:** Subscale scores on Thai version of the C19-YRS

Subscale	Range	Median
Symptom severity (0–90)	1–55	0
Functional disability (0–30)	0–12	0
Overall health (0–10)	0–10	9

There were significant correlations between the symptom severity, functional disability, and overall health subscales, as demonstrated in Table 3. There was a significant relationship between symptom severity and functional disability (r_s_=0.385, p value < 0.01). Furthermore, overall health was negatively correlated with symptom severity (r_s_ = − 0.291, p value < 0.01) and functional disability (r_s_ = − 0.108, p value < 0.05).

**Table 3:** Correlation of symptom severity, functional disability, and overall health

	Spearman’s rank correlation coefficient (*r*_s_)		
	Symptom severity	Functional disability	Overall health
Symptom severity	1.00		
Functional disability	0.385**	1.00	
Overall health	−0.291**	−0.108*	1.00

** p < .01, * *p* < .05

### Clusters of symptoms and functional disability

We examined items from two subscales of the Thai C19-YRS, symptom severity and functional ability. Seven items including breathlessness when dressing, swallowing difficulty, incontinence, anxiety, PTSD, personal care, and social roles were deleted because they did not meet the criteria for corrected item-total correlation [17]. The remaining 12 items were analyzed using EFA with Promax rotation. The KMO measure of sampling adequacy (0.818), Bartlett’s test of sphericity *(χ*^*2*^ = 2458.670, df = 66, *p* < .001), and MSA of each variable were greater than 0.5, indicating that the data set was appropriate for EFA.

**Fig. 2:**
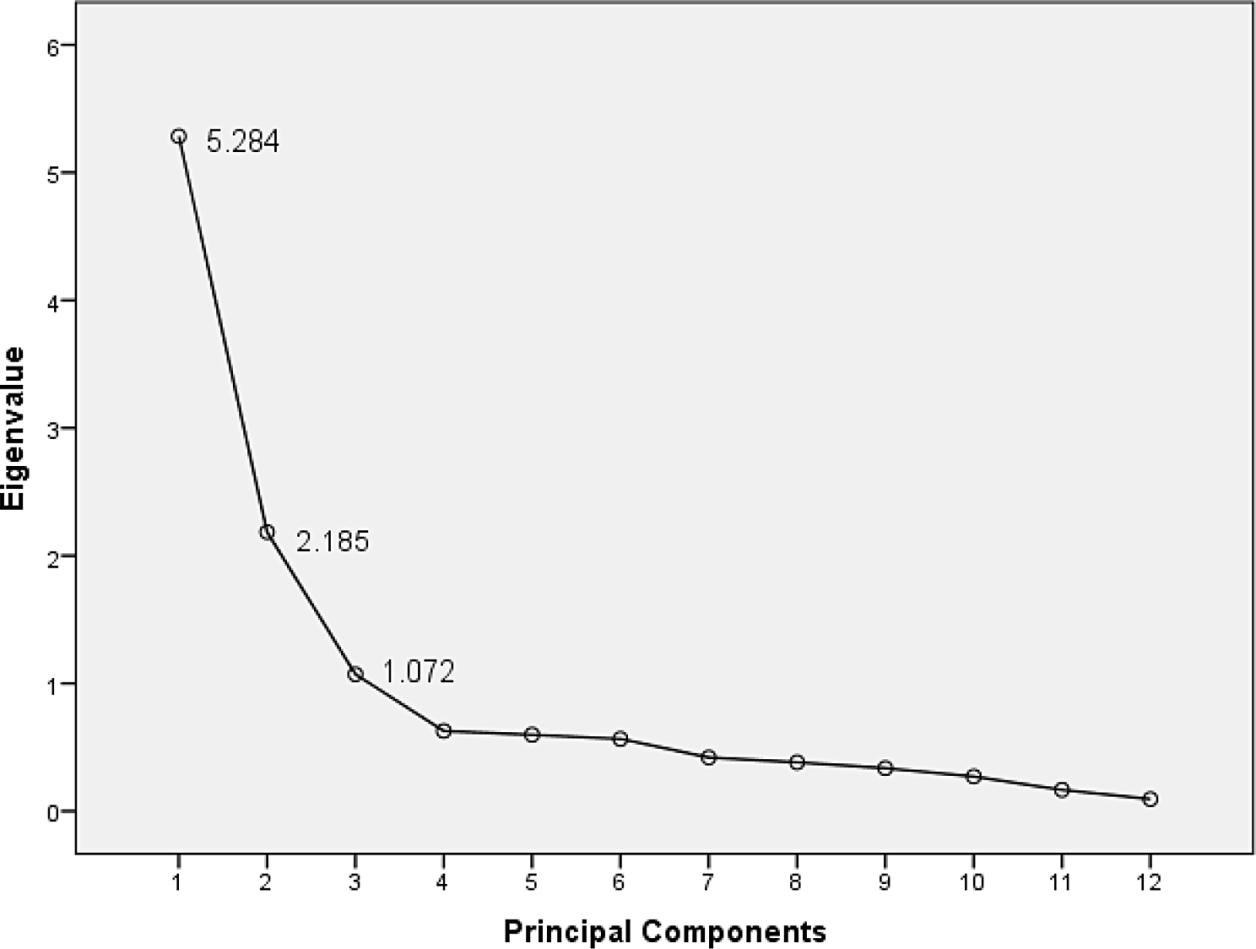
The Principal Component Analysis Scree Plot

The findings revealed three extracted factors with eigenvalues greater than 1 (Fig. 2), explaining 71.436% of the total variance. Eigenvalues, percent of variance, factor loadings, and communalities for each factor are presented in Supplementary Table 2.

Factor 1 included breathlessness at rest, cough or throat discomfort, communication, voice changes, and pain/discomfort and could explain 44.03% of the total variance with an eigenvalue of 5.284. This subscale was denoted “common symptoms of long COVID and communication.” The factor loadings ranged between 0.747 and 0.901, and the communalities ranged from 0.670 to 0.813.

Factor 2 incorporated mobility, activities of daily living, nutritional concerns, and fatigue and explained 18.207% of the total variance with an eigenvalue of 2.185. This factor was denoted “fatigue, functioning, and nutritional concerns.” The factor loadings ranged between 0.457 and 0.936, and the communalities ranged from 0.523 to 0.857.

Factor 3 comprised concentration, short-term memory, and depression and explained 8.929% of the total variance with an eigenvalue of 1.072. This factor was termed “psychosocial impacts.” The factor loadings ranged between 0.511 and 0.885 and the communalities ranged from 0.534 to 0.773.

## Discussion

The results of this study were based on the same data set as that in our recently published research on psychometric properties of the Thai version of C19-YRS [20]. In this research, we studied the prevalence of long COVID and the clusters of symptoms and disability among COVID-19 survivors in Thailand. We also presented the reduction of some items consistent with the changes in the original English version of the C19-YRS after our previous publication.

The prevalence of long COVID may vary worldwide depending on the SARS-CoV-2 variant causing infection, the region, and the time of data collection. The prevalence of long COVID declines over time post-acute infection [22, 23]. In this study, most participants (96.5%) had a post COVID-19 duration of more than 3 months. Of those, nearly 60% had long COVID for more than 6 months. Therefore, the persistence of long COVID was likely to be low in comparison with that of other studies. The prevalence (32.9%) in our study was lower than the global prevalence of long COVID, with prevalences of 43%, 51%, and 49% for the general population, the Asian population, and at 3 months after infection, respectively [24]. The reason for this might be that most participants who had mild symptoms (green category) during acute infection accounted for 88.1% of the study population, and 99.1% were not hospitalized. Furthermore, fewer than 20% of participants were in the older adult age group. Therefore, the prevalence of persistent symptoms would be lower than those of prior studies.

In the present study, we found that the two main symptoms of long COVID were anxiety (28.5%) and fatigue (26.1%), followed by dyspnea (13.4%). This could be related to findings from global studies on post COVID-19 syndrome reporting a prevalence for attention disorders of 27% [25], 23% for fatigue, and 13% for dyspnea [24]. Our study, with overcrowded communities and a low socioeconomic population. They may have financial problems resulting in limited access to healthcare services. These reasons could be related to the prevalence of anxiety, which was ranked the first problem in this study.

Being female was associated with a higher proportion of long COVID. This finding is supported by various studies [7, 10, 22–24, 26–28]. Females tend to have more severity than males, which may be due to the role of sex hormones influencing immune responses to infection. A discrepancy in the SARS-CoV-2 IgG antibody level in males and females at the early phase of infection may lead to different outcomes between the sexes [28]. Additionally, females are generally more concentrated on their bodies and related concerns [28]. Individuals with pre-existing conditions of obesity and cardiovascular disease were also more likely to develop symptoms of long COVID, which is in line with evidence in the literature [6, 7, 22, 26, 29]. The severity of symptoms during acute COVID was also related to symptoms of long COVID, which is similar to previous reports [24, 26].

The overall study population rated their symptom severity and functional disability using the C19-YRS as 0, representing the median. These findings were lower than those of other studies [18, 19, 27, 30]. The median score for overall health was 9 out of 10, which was correlated with mild symptom severity and lower impact on functional ability. Variations might be owing to differences in participants’ characteristics, different waves of the pandemic, the post COVID-19 duration, and differences in health care systems across regions. The results of this study supported those of previous studies [19, 30] in that we found significant relationships among symptom severity, functional disability, and overall health. Providing care for patients with long COVID should focus on both symptom management and rehabilitation to improve patient well-being.

The first factor cluster, “common symptoms of long COVID and communication,” comprised five items (breathlessness at rest, cough or throat discomfort, communication, voice changes, and pain or discomfort). Breathlessness upon walking up a flight of flight stairs was not loaded in any specific cluster, so it was not included in this study. Cough and changes in the voice are common respiratory problems in acute COVID and may last for many weeks. Cough and breathlessness occur as a result of SARS-CoV-2 targeting the respiratory tract and activating pro-inflammatory cytokines, thereby contributing to pulmonary vasculature damage [11, 12, 31]. Having these respiratory symptoms could limit one’s ability to communicate with others. Pain or discomfort could be defined as neuropathic pain and is correlated with increased levels of the cytokines, interleukin-6 and interleukin-10 [12]. These symptoms are frequently reported by patients with long COVID. This factor could explain approximately 44% of the total variance.

The second factor cluster, “fatigue, functioning, and nutritional concerns,” comprised four items (fatigue, mobility, activities of daily living, and nutritional concerns). As a CNS-mediated symptom of long COVID, fatigue is also caused by pro-inflammatory cytokines and is associated with decreased general activity, social interaction, and intake of food and water [12]. Poor food intake may lead to caloric deficit, which further contributes to fatigue. Therefore, fatigue syndrome, physical functioning, and nutritional problems should be assessed. Care management may need to cover all these related problems to prevent functional decline.

The third factor cluster, “psychosocial impact,” comprised three items (concentration, short-term memory, and depression). These three symptoms were loaded into one factor. SARS-CoV-2 could damage the CNS, contributing to neurocognitive and psychiatric disorders; therefore, these problems may share the same mechanism. Although this factor could explain approximately 9% of the total variance, how severity affects daily living should be further investigated, particularly in older adults. Anxiety is commonly found in patients with long COVID, although it is not included in the revised version of the C19-YRS owing to its internal consistency. This should be added to other symptoms on the scale, as recommended by Sivan et al. [17], to assess all mental conditions in post COVID syndrome.

Conceptually, it is reasonable to assume that the variables in each factor would be within the same factor because they share a common pathophysiology. Thus, designing a rehabilitation program to improve physical functioning among COVID-19 survivors should concurrently involve dietary management, promoting physical activity, and symptomatic treatment. Psychosocial problems should also be assessed and managed simultaneously.

## Limitations

The present study has some limitations. First, generalizability to the Thai population in other areas is limited because we only investigated one district in the capital city. Replication of the study in other regions is suggested. Second, owing to the cross-sectional research design, we did not follow individuals over time so the associations identified might be difficult to interpret. Recall bias may also be present. A cohort study to identify associations between long COVID symptoms and risk factors is recommended.

## Conclusion

The present study findings can help to fill knowledge gaps regarding the prevalence, symptom severity, functional disability, and overall health of COVID-19 survivors in Thailand. According to our study findings. We identified three clusters of major symptoms and functional disability using the Thai version of the C19-YRS, including: (1) “common symptoms of COVID-19 and communication,” (2) “fatigue, functioning, and nutritional concerns,” and (3) “psychosocial impacts.” These results could pave the way for effective management by multidisciplinary teams to lessen symptom severity, promote functional ability, and improve quality of life among individuals with long COVID. In terms of recommendations for future research, (1) rehabilitation programs by a multidisciplinary team for long COVID survivors should be researched in order to investigate their effectiveness; and (2) confirmatory factors analysis for symptoms of long COVID should be studied. Regarding health care policy, transforming the primary health care system by screening long COVID symptoms during the post-acute phase of infection in the communities should be proposed. Likewise, a referral system to a multidisciplinary team should be established promptly, with easy access to proper care in time.

## Electronic supplementary material

Below is the link to the electronic supplementary material.

Supplementary Material 1

## Data Availability

The data set will be available from the corresponding author after the reasonable request.

## Abbreviations

BMI: Body mass index
CI: Confidence interval
CNS: Central nervous system
COVID-19: Coronavirus disease
C19-YRS: COVID-19 Yorkshire Rehabilitation Scale
EFA: Exploratory factor analysis
KMO: Kaiser-Meyer-Olkin
MSA: Measure of sampling adequacy
OR: Odds ratio
PTSD: Post traumatic stress disorder
SARS-CoV-2: Severe acute respiratory syndrome coronavirus 2
THB: Thai Baht
