# Supplementary material for: Prevalence and exploratory factor analysis of long COVID-19 symptoms among experienced infected population in Bangkok, Thailand
Source: BMC Public Health. 2024 Oct 17;24:2863. doi: 10.1186/s12889-024-20275-5 (PMC11488197; doi:10.1186/s12889-024-20275-5)
Supplement: Supplementary file 1 — Supplementary Material 1 [file 12889_2024_20275_MOESM1_ESM.docx]

**Supplementary table 1** Characteristics of participants (n=337)

| **Participants’ characteristics** | **n (%)** |
| --- | --- |
| *Age, years* (mean=45.94, SD=15.61) |  |
| 21–60 | 278 (82.5) |
| >60 | 59 (17.5) |
| *Sex* |  |
| Female | 181 (53.7) |
| Male | 156 (46.3) |
| *Education* |  |
| No education | 22 (6.5) |
| Primary education | 131 (38.9) |
| Secondary education | 138 (40.9) |
| Bachelor’s degree | 43 (12.8) |
| Master’s degree | 3 (0.9) |
| *Occupation* |  |
| Unemployed | 78 (23.1) |
| Employed | 100 (29.7) |
| Sales | 67 (19.9) |
| Governor | 6 (1.8) |
| State enterprise employee | 7 (2.1) |
| Private company employee | 63 (18.7) |
| Business owner | 11 (3.3) |
| Student | 5 (1.5) |
| *Family income* (THB/month) |  |
| ≤10,000 | 109 (32.4) |
| 10,001–20,000 | 148 (43.9) |
| 20,001–30,000 | 50 (14.8) |
| 30,001–40,000 | 18 (5.3) |
| >40,000 | 12 (3.6) |
| *Body mass index* (BMI, kg/m^2^) |  |
| Underweight (<18.5) | 18 (5.3) |
| Normal weight (18.5–22.9) | 107 (31.8) |
| Overweight (23-24.9) | 61 (18.1) |
| Obese (≥25) | 151 (44.8) |
| *Past medical history* |  |
| No underlying disease | 191 (56.7) |
| Having underlying disease | 146 (43.3) |
| Asthma | 5 (1.5) |
| Diabetes | 40 (11.9) |
| Hypertension | 81 (24) |
| Heart disease | 13 (3.9) |
| Stroke | 7 (2.1) |
| Cancer | 3 (0.9) |
| *Underlying cardiovascular disease* |  |
| No | 233 (69.14) |
| Yes | 104 (30.86) |
| *Current smoking* |  |
| No | 276 (81.9) |
| Yes | 61 (18.1) |
| *Symptom categories during acute COVID-19 infection* |  |
| Mild (green) | 297 (88.1) |
| Moderate (yellow) | 29 (8.6) |
| Severe (red) | 11 (3.3) |
| *Developed pneumonia during acute COVID* |  |
| No | 277 (82.2) |
| Yes | 60 (17.8) |
| *Acute care setting* |  |
| Home isolation | 111 (32.9) |
| Field hospital | 196 (58.2) |
| Ward | 28 (8.3) |
| Intensive care unit | 2 (0.6) |
| *Post COVID-19 duration* (months) |  |
| <3 | 12 (3.6) |
| 3–6 | 133 (39.5) |
| >6 | 192 (57.0) |
| *Symptoms of long COVID* |  |
| No symptoms | 226 (67.1) |
| At least one symptom | 111 (32.9) |

SD, standard deviation.

**Supplementary table 2** Exploratory factor analysis of Thai C19-YRS (n=337)

|  | Item | Factor loadings | Communalities (h^2^) |
| --- | --- | --- | --- |
| **Factor 1 Common symptoms of long COVID and communication** (5 items)  Eigenvalue=5.284; percent of variance=44.030 | | | |
| 1 | How breathless do you feel at rest? (Breathlessness at rest) | 0.901 | 0.704 |
| 2 | Have you developed any changes in the sensitivity of your throat such as a troublesome cough or noisy breathing? (Cough or throat discomfort) | 0.830 | 0.670 |
| 13 | Have you or your family noticed any changes in the way you communicate with people, such as understanding what people say to you, translating your messages from thoughts into words, or difficulties reading or having a conversation? (Communication) | 0.828 | 0.813 |
| 3 | Have you or your family noticed any changes in your voice such as others having difficulty hearing your voice, changes in the quality of your voice, low voice by the end of the day, or an inability to alter the pitch of your voice? (Changes in voice) | 0.799 | 0.743 |
| 11 | How would you rate the level of your pain or discomfort? (Pain or discomfort) | 0.747 | 0.704 |
| **Factor 2 Fatigue, functioning, and nutritional concerns** (4 items)  Eigenvalue=2.185; percent of variance=18.207 | | | |
| 6 | Have you experienced difficulty walking? (Mobility) | 0.936 | 0.841 |
| 10 | How would you rate the level of difficulty in doing your activities, such as your household duties, leisure activities, work, or study? (Activities of daily living) | 0.895 | 0.857 |
| 5 | Do you or your family have concerns about persistent weight loss or nutritional problems caused by COVID-19 symptoms? (Nutritional concerns) | 0.753 | 0.690 |
| 7 | Are you more easily exhausted now than you were before your illness? (Fatigue) | 0.457 | 0.523 |
| **Factor 3 Psychosocial impact** (3 items)  Eigenvalues= 1.072; percent of variance=8.929 | | | |
| 12.1 | Since you became ill, have you had any new problems or worse conditions regarding concentration? (Concentration) | 0.885 | 0.773 |
| 12.2 | Since you became ill, have you had any new problems or worse conditions in terms of short-term memory? (Short-term memory) | 0.760 | 0.687 |
| 15 | How would you rate the severity of your depression? (Depression) | 0.511 | 0.534 |
